# Supplementary material for: A spectral framework for measuring diversity in multiple sequence alignments
Source: PLoS Comput Biol. 2026 Sep 23;22(9):e1014778. doi: 10.1371/journal.pcbi.1014778 (PMC13619056; doi:10.1371/journal.pcbi.1014778)
Supplement: Supplementary file 1 [file pcbi.1014778.s001.pdf]

Supporting information: A spectral framework for measuring diversity in multiple sequence alignments

Vaitea OPUU[a,‡]

[a]Laboratoire de Biophysique et Evolution, UMR CNRS-ESPCI 8231 Chimie Biologie Innovation, PSL University, France,
‡vaitea.opuu@espci.fr

Sampling sequences from a multivariate Gaussian model

How I sampled sequences from a covariance matrix C. C is the covariance matrix of a one-hot encoded MSA. To sample sequences from this distribution assuming an average symbol distribution $\mu = (1/k, ...1/k)$. First, we compute the eigenvalues and eigenvectors (λ_i, v_i) of C. The second step is to sample a novel sequence $x = \sum_i v_i(\lambda_i \dot{\epsilon})$, where $\epsilon \sim \mathcal{N}(0, 1)$. The last step consists in converting the x vector into a one hot encoded sequence with nearest-neighbor approach which assigns the symbol identity of the closest one hot encoded nucleotide. Since we assume a uniform distribution of the symbols, the positional perplexity is always maximized, regardless of the interaction level encoded in the covariance matrix.

Direct Coupling Analysis

Direct Coupling Analysis (DCA) infers residue–residue interactions by fitting a pairwise maximum-entropy (Potts) model that reproduces the empirical single-site and pairwise frequencies observed in a multiple sequence alignment [1]. The statistical model is

$$P(s) = \frac{1}{Z} \exp\left(\sum_i h_i(s_i) + \sum_{i<j} J_{ij}(s_i, s_j) \right),$$

where $s = (s_1, \ldots, s_L)$ denotes a sequence, h_i are site-specific fields, J_{ij} are direct couplings, and Z is the partition function. The inferred couplings quantify conditional dependencies that remain after removing indirect correlations, enabling high-resolution contact prediction and structural inference. Extensions such as eaDCA have been applied to estimate the effective support size of high-dimensional sequence distributions [2], and DCA-based generative models have been used for rational sequence design, including the *in silico* design of functional RNAs [3]. To run DCA (fully connected), I adapted the python code from [4] which runs Gibbs Markov Chain Monte Carlo for 100 sweeps. We checked the convergence by comparing pairwise frequencies between a 1000 MCMC sample and the MSA.

Variational autoencoder

The variational autoencoder (VAE) provides a generative model for RNA sequence families by learning a latent representation from multiple sequence alignments [5]. Each sequence is mapped to a continuous latent variable drawn from a Gaussian distribution, and a neural decoder transforms this latent vector into position-wise categorical probabilities over the nucleotide alphabet. Training maximizes the evidence lower bound, combining a reconstruction term with a KL regularizer that encourages the inferred latent distribution to remain close to a standard normal prior [6]. To train the models, I ran 100 epochs (with batch size 32) of learning using the Adam optimizer [7]. After training, new sequences are generated by sampling latent vectors from the prior, $z \sim \mathcal{N}(0, \sigma^2 I)$, and decoding them into categorical logits at each sequence position; discrete sequences are then produced by selecting the most likely nucleotide at each site. For our experiments, we typically generate 1000 sequences.

Nearest neighbor distance

For each generated sequence, I compute its maximum identity to any training sequence, normalized by its maximum identity to any other sequence in the generated library. Averaging over the library gives:

$$\text{NN}(X, \text{TRAIN}) = \frac{1}{|X|} \sum_{x \in X} \frac{\max_{y \in \text{TRAIN}} \text{id}(x, y)}{\max_{\substack{x' \in X \\ x' \neq x}} \text{id}(x, x')} \quad (1)$$

where $\text{id}(x, y)$ is the sequence identity between two sequences. A value near 1 means generated sequences are no more similar to training examples than to each other; values above 1 indicate on-manifold generation clustered near known functional sequences, whereas values below 1 indicate off-manifold exploration.

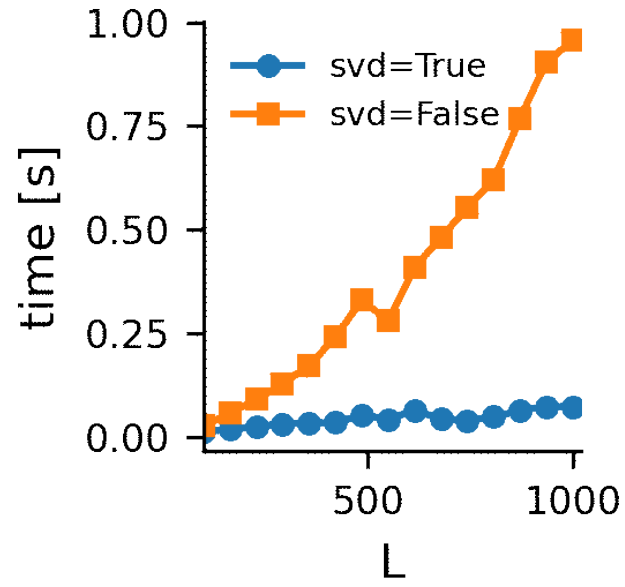

Fig A: Runtime of the effective length computation as a function of sequence length L for fixed sample size $N = 100$ and alphabet size $k = 4$. Synthetic MSAs are generated from a Gaussian covariance model with correlation parameter $\rho = 0.5$. Execution time is reported for two implementations: singular value decomposition and covariance eigen-decomposition. Each point corresponds to the mean wall–clock time over independent realizations.

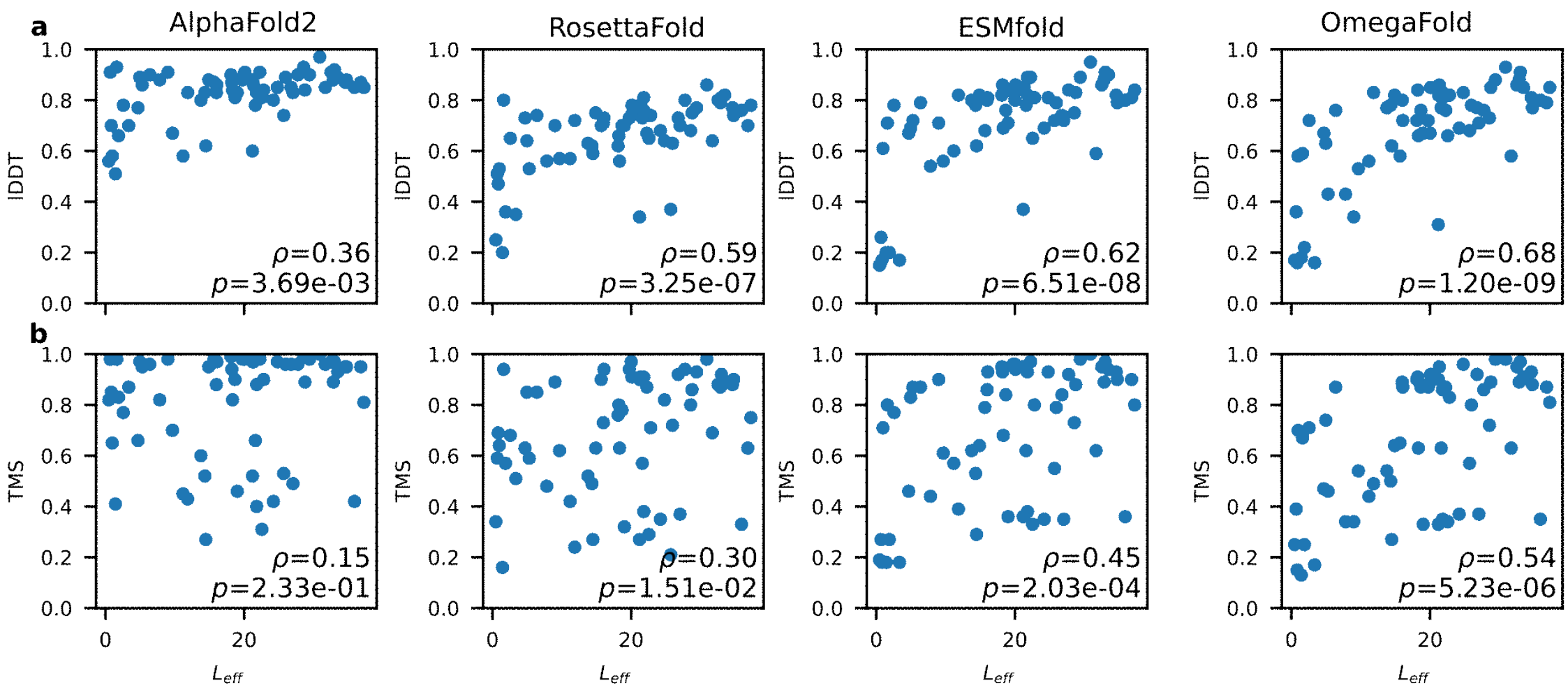

Fig B: Relationship between sequence diversity (L_{eff}) and protein structure prediction quality. **(a)** Scatter plots showing the correlation between the effective length (L_{eff}) and the Local Distance Difference Test (LDDT) for four transformer-based models: AlphaFold2, RoseTTAFold, ESMFold, and OmegaFold. Spearman correlation coefficients (ρ) and p-values are shown for each model, with single-sequence PLM-based models (ESMFold and OmegaFold) exhibiting the strongest dependencies on family diversity. **(b)** Corresponding correlations for the global TM-score. All benchmarks were performed on the CASP15 dataset [8].

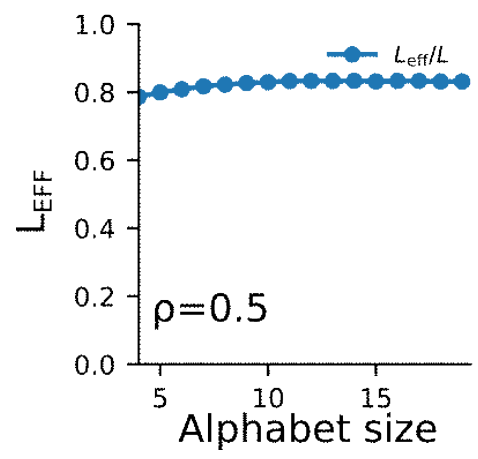

Fig C: Alphabet size effect on L_{eff}. Synthetic MSAs of size N=500 and $\rho = 0.5$ but alphabet size k is varying from 4 to 20. The L_{eff} is recorded and shown in blue.

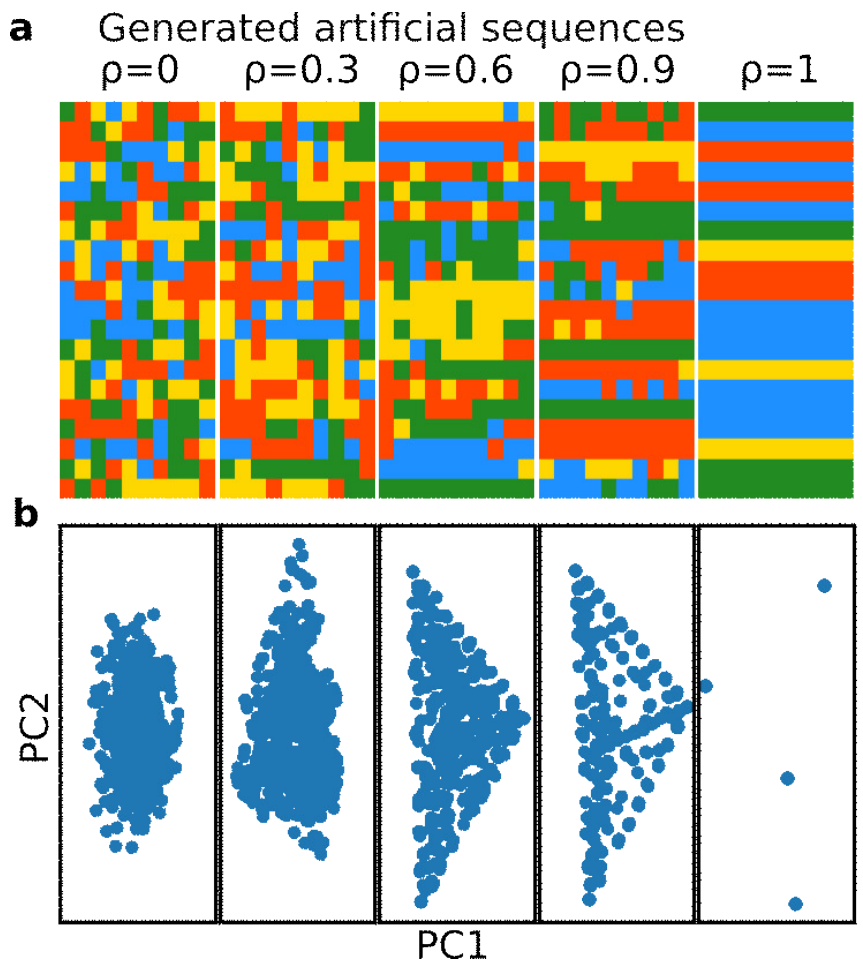

Fig D: Synthetic MSA with varying correlation strength between positions and symbols. In the first row (a), I show the MSA, where one colour corresponds to a nucleotide and each row is a sequence. In the second row (b), I show the PCA projection of the MSA, showing how they converge to four typical sequences.

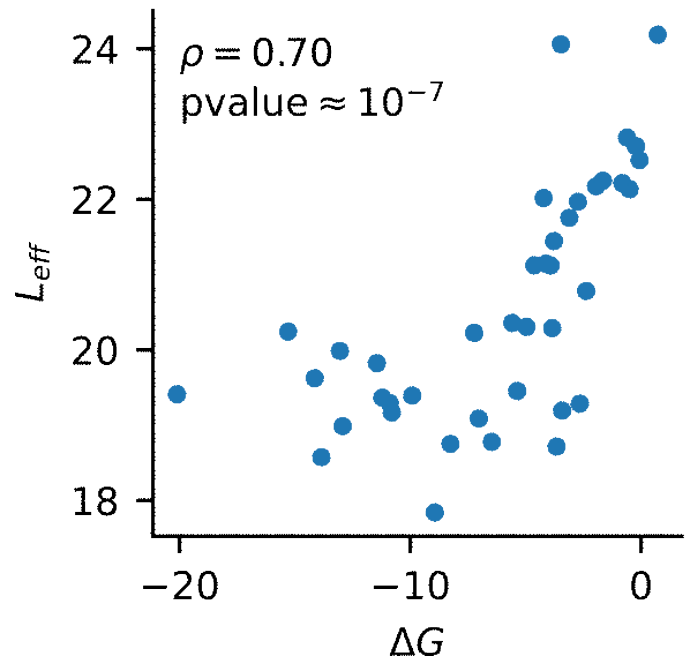

Fig E: Folding ΔG correlates with L_{eff}. I generated 500 variants using RNAinverse on 40 randomly generated structures. I computed L_{eff} for each of the 40 datasets together with the average folding stability (computed with ViennaRNA [9]), which is shown as a scatter plot.

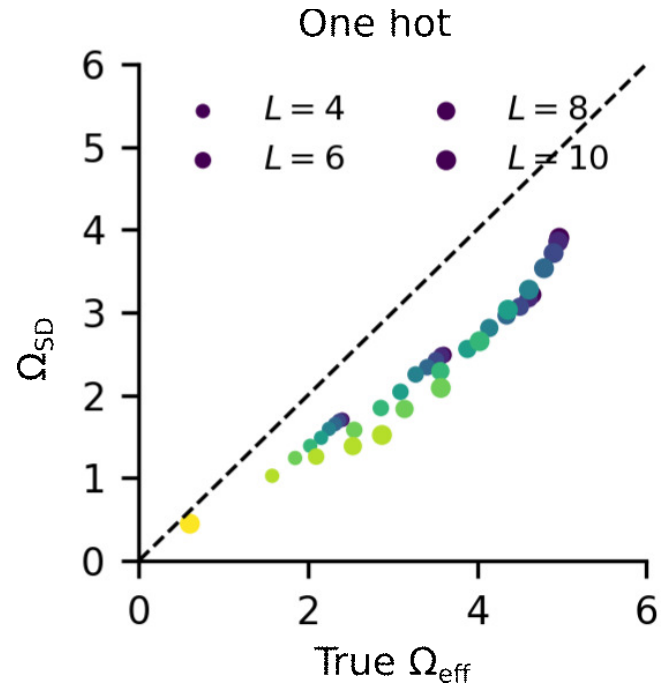

Fig F: Scatter plot of the estimated effective support size ($\Omega_{eff} = k^{L_{eff}}$) using directly the one hot encoding instead of the Helmert encoding, colored by sequence length L (4 to 10). The dashed line represents the identity function ($y = x$).

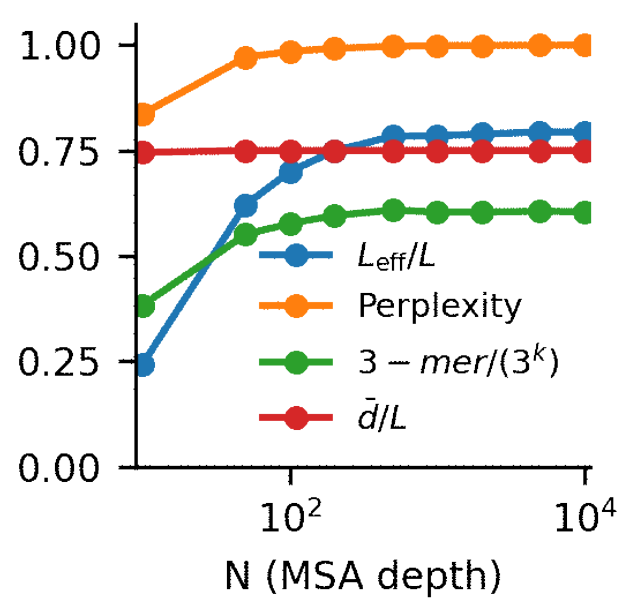

Fig G: Effect of MSA depth on diversity estimates. Synthetic MSAs generated with fixed correlation ($\rho = 0.5$) are analyzed as a function of sample size N. Entropy-based measures (positional entropy and k-mer entropy) vary with depth, whereas average pairwise distance remains largely insensitive.

References

[1] Faruck Morcos, Andrea Pagnani, Bryan Lunt, Arianna Bertolino, Debora S Marks, Chris Sander, Riccardo Zecchina, José N Onuchic, Terence Hwa, and Martin Weigt. Direct-coupling analysis of residue coevolution captures native contacts across many protein families. *Proceedings of the National Academy of Sciences*, 108(49):E1293–E1301, 2011.

[2] Francesco Calvanese, Camille N Lambert, Philippe Nghe, Francesco Zamponi, and Martin Weigt. Towards parsimonious generative modeling of rna families. *Nucleic Acids Research*, 52(10):5465–5477, 2024.

[3] Camille N Lambert, Vaitea Opuu, Francesco Calvanese, Polina Pavlinova, Francesco Zamponi, Eric J Hayden, Martin Weigt, Matteo Smerlak, and Philippe Nghe. Exploring the space of self-reproducing ribozymes using generative models. *Nature Communications*, 16(1):7836, 2025.

[4] Anna Paola Muntoni, Andrea Pagnani, Martin Weigt, and Francesco Zamponi. adabmdca: adaptive boltzmann machine learning for biological sequences. *BMC bioinformatics*, 22(1):528, 2021.

[5] Alex Hawkins-Hooker, Florence Depardieu, Sebastien Baur, Guillaume Couairon, Arthur Chen, and David Bikard. Generating functional protein variants with variational autoencoders. *PLoS computational biology*, 17(2):e1008736, 2021.

[6] Diederik P Kingma and Max Welling. Auto-encoding variational bayes. *arXiv preprint arXiv:1312.6114*, 2013.

[7] Diederik P Kingma. Adam: A method for stochastic optimization. *arXiv preprint arXiv:1412.6980*, 2014.

[8] Bernard Moussad, Rahmatullah Roche, and Debswapna Bhattacharya. The transformative power of transformers in protein structure prediction. *Proceedings of the National Academy of Sciences*, 120(32):e2303499120, 2023.

[9] Ronny Lorenz, Stephan H Bernhart, Christian Höner zu Siederdissen, Hakim Tafer, Christoph Flamm, Peter F Stadler, and Ivo L Hofacker. Viennarna package 2.0. *Algorithms for molecular biology*, 6(1):26, 2011.
